# The RNase J-Based RNA Degradosome Is Compartmentalized in the Gastric Pathogen Helicobacter pylori

**DOI:** 10.1128/mBio.01173-20

**Published:** 2020-09-15

**Authors:** Alejandro Tejada-Arranz, Eloïse Galtier, Lamya El Mortaji, Evelyne Turlin, Dmitry Ershov, Hilde De Reuse

**Affiliations:** a Institut Pasteur, Département de Microbiologie, Unité Pathogenèse de Helicobacter, UMR CNRS 2001, Paris, France; b Université de Paris, Sorbonne Paris Cité, Cellule Pasteur, Paris, France; c Image Analysis Hub, C2RT, Institut Pasteur, Paris, France; d Bioinformatics and Biostatistics HUB, Department of Computational Biology, Institut Pasteur, USR 3756 CNRS, Paris, France; Max Planck Unit for the Science of Pathogens

**Keywords:** *Helicobacter pylori*, RNA degradosome, RNase J, post-transcriptional regulation, subcellular localization, superresolution microscopy

## Abstract

Posttranscriptional regulation is a major level of gene expression control in any cell. In bacteria, multiprotein machines called RNA degradosomes are central for RNA processing and degradation, and some were reported to be compartmentalized inside these organelleless cells. The minimal RNA degradosome of the important gastric pathogen Helicobacter pylori is composed of the essential ribonuclease RNase J and RhpA, its sole DEAD box RNA helicase, and plays a major role in the regulation of mRNA decay and adaptation to gastric colonization. Here, the subcellular localization of the H. pylori RNA degradosome was investigated using cellular fractionation and both confocal and superresolution microscopy. We established that RNase J and RhpA are peripheral inner membrane proteins and that this association was mediated neither by ribosomes nor by RNA nor by the RNase Y membrane protein. In live H. pylori cells, we observed that fluorescent RNase J and RhpA protein fusions assemble into nonpolar foci. We identified factors that regulate the formation of these foci without affecting the degradosome membrane association. Flotillin, a bacterial membrane scaffolding protein, and free RNA promote focus formation in H. pylori. Finally, RNase J-GFP (RNase J-green fluorescent protein) molecules and foci in cells were quantified by three-dimensional (3D) single-molecule fluorescence localization microscopy. The number and size of the RNase J foci were found to be scaled with growth phase and cell volume as previously reported for eukaryotic ribonucleoprotein granules. In conclusion, we propose that membrane compartmentalization and the regulated clustering of RNase J-based degradosome hubs represent important levels of control of their activity and specificity.

## Abstract (precis)

Helicobacter pylori is a bacterial pathogen that chronically colonizes the stomach of half of the human population worldwide. Infection by H. pylori can lead to the development of gastric pathologies such as ulcers and adenocarcinoma, which causes up to 800,000 deaths in the world each year. Persistent colonization by H. pylori relies on regulation of the expression of adaptation-related genes. One major level of such control is posttranscriptional regulation, which, in H. pylori, largely relies on a multiprotein molecular machine, an RNA degradosome, that we previously discovered. In this study, we established that the two protein partners of this machine are associated with the membrane of H. pylori. Using cutting-edge microscopy, we showed that these complexes assemble into hubs whose formation is regulated by free RNA and scaled with bacterial size and growth phase. Organelleless cellular compartmentalization of molecular machines into hubs emerges as an important regulatory level in bacteria.

## INTRODUCTION

Posttranscriptional regulation is one of the most important levels of control of gene expression in every kingdom of life. Ribonucleases (RNases) are key enzymes in posttranscriptional regulation, involved in RNA maturation and degradation. RNases often act in multiprotein complexes that are designated exosomes in Eukarya and Archaea and RNA degradosomes in bacteria and chloroplasts (for a review, see reference 1). RNA degradosomes are defined by two core components, an RNase and an RNA helicase of the DEAD box family (1, 2). Most RNA degradosomes reported so far are assembled on the endoribonuclease RNase E, as seen in Escherichia coli, Caulobacter crescentus, or Mycobacterium tuberculosis (3–5). Nevertheless, RNase E is absent from about half of the bacterial species, and most (47%) of the remaining bacteria have RNase Y enzymes or RNase J enzymes or both (1). RNase J and RNase Y both display endoribonucleolytic activities, and RNase J acts in addition as a 5′–3′ exoribonuclease (6, 7). In the Gram-positive bacteria Staphylococcus aureus and Bacillus subtilis, RNA degradosomes comprising RNase Y and RNase J have been proposed (8, 9), but to date their functionality has not been clearly established, and in B. subtilis, the complex was detected only after cross-linking (10).

In the Gram-negative pathogen Helicobacter pylori, we recently demonstrated the existence of a minimal RNA degradosome composed of two partners (11–13). H. pylori is a spiral-shaped bacterium that persistently colonizes the stomach of more than half of the human population worldwide (14) and causes chronic gastritis, peptic ulcers, mucosa-associated lymphoid tissue (MALT) lymphoma, and gastric carcinoma, which is responsible for 800,000 deaths per year in the world (15, 16). H. pylori possesses a small genome (1.6 Mb) and a reduced number of transcriptional regulators. Therefore, posttranscriptional regulation has been proposed to play a major role in the adaptive response of H. pylori (17).

The RNA degradosome of H. pylori is composed of the essential RNase J protein and of RhpA, its sole DEAD box RNA helicase (11–13). *In vitro*, each protein of this complex stimulates the activity of the other, demonstrating that they form a functional RNA degradosome (11). Moreover, both proteins were detected in association with translating ribosomes, suggesting a coupling between translation and RNA degradation (11). In an H. pylori RNase J-depleted strain, the amounts of 55% of mRNAs and 40% of antisense RNAs (asRNAs) are increased more than 4-fold in the mutant in comparison with the parental wild-type strain (12), demonstrating a major role of RNase J in mRNA decay in H. pylori.

The RNase activities of the RNA degradosomes are generally essential for bacterial survival, but they are also potentially destructive if they degrade RNAs that are important for growth. Accordingly, several levels of control of degradosomes have been identified, including compartmentalization (1). Emerging compartmentalized structures are associated with membranes or are the so-called membraneless organelles formed by liquid-liquid-phase separation (LLPS). The RNase E-based degradosome of E. coli localizes at the inner membrane of the cell (18, 19), and E. coli RNase E (*Eco*RNase E) fluorescent fusion proteins form short-lived foci and rapidly diffuse across the inner membrane (18, 20). In C. crescentus, RNase E (*Cc*RNase E) lacks a membrane anchor and is cytoplasmic (21). *Cc*RNase E-YFP fusions form clusters that are dynamically assembled, change with cell cycle or stress exposure, and are proposed to be RNA-cleavage sites (22, 23). These foci present characteristics of LLPS similar to those seen with eukaryotic messenger ribonucleoprotein (mRNP) granules, such as p-bodies or stress granules (1). In S. aureus and B. subtilis, the proposed RNA degradosomes rely on the membrane-anchored RNase Y (24–26). In B. subtilis, an RNase Y-GFP (RNase Y-green fluorescent protein) fusion was observed to have a homogeneous membrane localization (25) and was recently found to be dynamic and to form foci similar to those of RNase E (27). Under conditions where RNase Y was less active, the foci were more abundant and showed increased size, suggesting that these structures represent a less active form of the enzyme (27). RNases J1 and J2 were found to localize in the cytoplasm and were excluded from the nucleoid (25). In S. aureus, FloA, a membrane scaffolding protein homologous to eukaryotic flotillin, promotes the oligomerization and the activity of RNase Y (28).

Thus, compartmentalization seems to be a frequent feature of bacterial RNA degradomes that is subjected to regulation and that probably controls their activity. Therefore, the subcellular localization of RNA degradosomes is an important question that was only addressed for a few degradosomes. Here, this question was addressed using the major pathogen H. pylori as a model organism.

In this report, we show that both H. pylori RNase J and RhpA target the inner membrane and form foci. We demonstrated that RNase J foci are subject to variations as a function of growth phase and antibiotic exposure and in different mutants, suggesting differential regulation of the activity of the RNA degradosome between focus-forming and non-focus-forming forms.

## RESULTS

### Localization of RNase J and RhpA at the H. pylori inner membrane.

**FIG 1:**
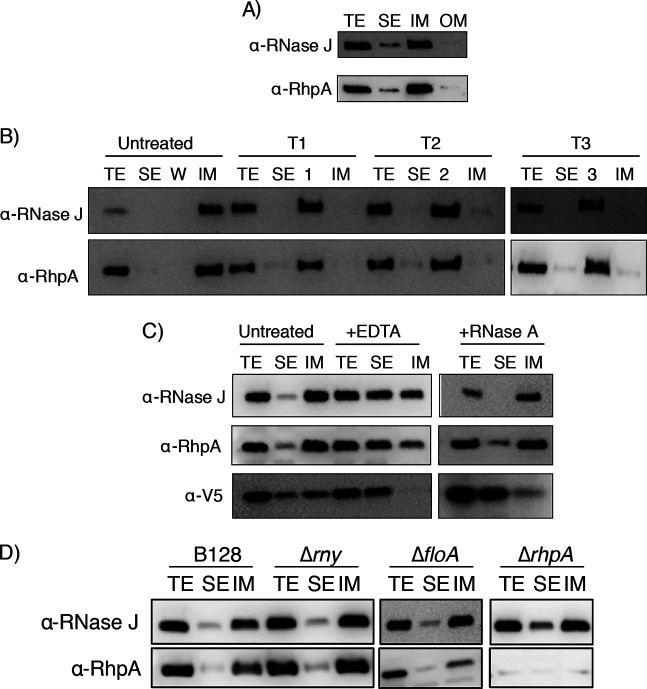
The two partners of the RNA degradosome of H. pylori, RNase J and RhpA, are associated with the inner membrane in a peripheral manner, independently of ribosomes and RNA, and this association does not depend on RNase Y, flotillin, or RhpA. Cellular compartments of H. pylori B128 strain were separated by fractionation; all samples correspond to the same initial number of bacteria. Experiments were performed in triplicate. TE, total extract fraction; SE, soluble extract fraction; IM, inner membrane fraction; OM, outer membrane fraction. (A) Western blot with antibodies against RNase J and RhpA on the different cellular fractions of wild-type (WT) H. pylori strain B128. (B) Western blot with antibodies against RNase J and RhpA on the different cellular fractions of WT H. pylori upon treatment of the membrane fraction with 6 M urea (T1), 100 mM Na_2_CO_3_ (T2), or 2 M NaCl (T3); untreated fractions are shown as a control. Lanes 1, 2, and 3 indicate the wash fractions corresponding to treatments T1, T2, and T3, respectively. (C) Western blot with antibodies against RNase J and RhpA and a V5 tag (marking the L9 ribosomal protein) on the different cellular fractions of an L9-V5-expressing H. pylori strain, under untreated conditions and upon treatment with 20 mM EDTA or 1 μg/ml RNase A. (D) Western blot with antibodies against RNase J and RhpA on the different subcellular fractions of WT, Δ*rny*, Δ*floA*, and Δ*rhpA* H. pylori strains.

The subcellular localization of RNase J and RhpA was determined in H. pylori strain B128 using a cellular fractionation protocol adapted to this bacterium according to a previously described method (29). Separation of the different fractions was validated using control antibodies, including anti-BabA for the outer membrane (OM), anti-MotB for the inner membrane (IM), and anti-AmiE for the soluble extract (SE) (Fig. S1A). Western blotting using specific anti-RNase J and anti-RhpA antibodies (11) revealed that both proteins are mostly present at the inner membrane, with a small amount in the soluble fraction (Fig. 1A). No protein was detected in the outer membrane. The same result was obtained with another H. pylori strain, the first-sequenced and well-studied 26695 strain (Fig. S1B).

10.1128/mBio.01173-20.1 FIG S1 (A) Western blots with antibodies against AmiE (soluble extract fraction protein), MotB (inner membrane fraction protein), and BabA (outer membrane fraction protein) on samples subjected to cellular fractionation. (B) Western blots with antibodies against RNase J and RhpA of H. pylori strain 26695 on samples subjected to cellular fractionation. TE, total extract fraction; SE, soluble extract fraction; IM, inner membrane fraction; OM, outer membrane fraction. (C) Prediction of RNase J transmembrane domains by the TMHMM, PureseqTM, and TMPred algorithms and of intrinsically disordered regions (IDRs) by the IUPred2A algorithm. (D) Prediction of RhpA transmembrane and intrinsically disordered regions performed as described for panel C. (E) Prediction of RNase Y transmembrane regions performed as described for panel C. Download FIG S1, TIF file, 2.3 MB. Copyright © 2020 Tejada-Arranz et al. 2020 Tejada-Arranz et al. This content is distributed under the terms of the Creative Commons Attribution 4.0 International license.

### RNase J and RhpA are peripheral membrane proteins.

Since no transmembrane domains are predicted in the sequences of RNase J and RhpA (Fig. S1C and D), we investigated the nature of the association of the RNA degradosome with the membrane. For this purpose, different treatments were applied to the purified membrane fractions, which were subsequently subjected to ultracentrifugation to separate the supernatant, which contained the extracted peripheral membrane proteins, from the pellet, which contained the integral membrane proteins. These treatments included the use of (i) 6 M urea, a chaotropic agent that weakens the hydrophobic interactions without disrupting the lipid bilayer; (ii) alkaline pH (100 mM Na_2_CO_3_; pH 11); and (iii) a high salt concentration (2 M NaCl). The three treatments consistently dissociated both RhpA and RNase J from the pelleted membrane fraction (Fig. 1B), clearly indicating that these two proteins are associated with the membrane through electrostatic and hydrophobic interactions and are thus peripheral inner membrane proteins in H. pylori.

### Membrane targeting of the degradosome is independent of ribosomes and RNA.

Our previous work revealed the association of at least a fraction of the H. pylori degradosome proteins with translating ribosomes (11). To examine whether the ribosomes might have been copurified with the inner membrane fraction and/or whether the RNase J and RhpA membrane association could be mediated by ribosomes, we constructed an H. pylori strain expressing a fusion between the L9 ribosomal protein and a V5 epitope from the native locus at the chromosome (Fig. S2A), which we used as a reporter for ribosome localization. Using this strain, we observed that, upon fractionation, the ribosomes were approximately evenly distributed between the inner membrane and soluble fractions (Fig. 1C). Treatment with EDTA, which, through Mg^2+^ chelation, causes dissociation of the ribosomal subunits, resulted in a complete delocalization of L9-V5 from the membrane fraction. EDTA treatment only partially delocalized RNase J and RhpA toward the soluble extract (Fig. 1C). Given that EDTA acts not only on ribosomes but also on the divalent cation pool of the cell and, consequently, on electrostatic interactions, it is difficult to dissociate the two effects in the analysis of this localization. To clarify the nature of the effects, we performed a treatment with RNase A, which degrades all unprotected RNAs and therefore dissociates polysomes. This treatment resulted in detachment of most of the ribosomes from the inner membrane. Importantly, under these conditions, neither RNase J nor RhpA delocalized toward the soluble extract, which strongly suggests that their membrane interaction is not mediated by ribosomes or by RNA and that it is, at least in part, cation dependent (Fig. 1C). Thus, ribosomes and RNA are not required for the inner membrane localization of the two proteins of the H. pylori degradosome and divalent cations are most probably important for their electrostatic membrane binding.

10.1128/mBio.01173-20.2 FIG S2 (A) Schematic representation of the genetic organization of the chromosomal regions of strain B128 expressing L9-V5, RNase J-GFP, RhpA-CFP, RNase J-FLAG, and RhpA-FLAG in the corresponding strains. KanaR, kanamycin resistance cassette; ApraR, apramycin resistance cassette; CmR, chloramphenicol resistance cassette. (B) Representative phase-contrast microscopy images of the B128 wild type and strains expressing RNase J-GFP or RhpA-CFP. No difference in morphology is observed between these strains. (C) Western blots with antibodies against RNase J and RhpA on samples subjected to cellular fractionation of strains expressing or not expressing RNase J-GFP or RhpA-CFP. TE, total extract fraction; SE, soluble extract fraction; IM, inner membrane fraction. (D) Representative confocal fluorescence microscopy images of H. pylori B128 strains overexpressing GFP (left) or CFP (right) alone from plasmid pILL2157. Fluorescence is distributed all over the cell. (E) Immunofluorescence confocal microscopy of fixed H. pylori cells expressing RNase J-FLAG (left) and RhpA-FLAG (right) with primary antibodies against the FLAG-tag and secondary anti-mouse AF488 antibody. (F) The RNase J-GFP foci remain static inside the cells. The displacement of foci was analyzed during 175 min either in live bacteria (*n* = 285 foci) or in fixed bacteria (*n* = 696 foci) as a control. The small change measured in the live bacteria is below the pixel area and thus nonsignificant. MSD, mean square displacement. Download FIG S2, PDF file, 1.0 MB. Copyright © 2020 Tejada-Arranz et al. 2020 Tejada-Arranz et al. This content is distributed under the terms of the Creative Commons Attribution 4.0 International license.

### Membrane targeting of the degradosome is independent of RNase Y and flotillin.

We wanted to explore the possibility that RNase J and RhpA could be indirectly bound to the membrane through another partner. In B. subtilis, RNase J1 was reported to be associated with the membrane-bound RNase Y. However, the main localization of B. subtilis RNase J1 seems to be cytoplasmic (25). A transmembrane domain is also predicted in the sequence of the H. pylori RNase Y protein (Fig. S1E). Therefore, an H. pylori mutant carrying a deletion of the corresponding *rny* gene was constructed and used to examine the impact of its absence on the localization of the degradosome. As shown in Fig. 1D, in this mutant, both RNase J and RhpA were still associated with the inner membrane, indicating that RNase Y is not the membrane anchor of the degradosome in H. pylori.

It has also been shown that flotillin, a bacterial membrane scaffolding protein related to the flotillins from eukaryotic lipid rafts, plays a role in the oligomerization state of S. aureus RNase Y, a component of the RNA degradosome in this organism (28). H. pylori possesses only one flotillin, which was recently identified (30). The corresponding *floA* gene (HPB128_21g23 in strain B128) is located immediately downstream of the *rhpA* gene (Fig. S2A). We found that the inner membrane association of the RNase J and RhpA proteins was not affected in a Δ*floA* mutant compared to the wild-type strain and thus that flotillin is not required for their targeting to the membrane (Fig. 1D).

We also wanted to test the respective roles of each protein in the membrane localization of its partner. Since RNase J is an essential protein, only a *ΔrhpA* mutant could be examined. We observed that RhpA is not required for the membrane targeting of RNase J. Under these conditions, the larger amount of RNase J present in the soluble extract was attributed to its overexpression in the *ΔrhpA* mutant, as we previously reported (13) (Fig. 1D).

### RNase J-GFP and RhpA-CFP form foci in live H. pylori cells.

We then wanted to analyze the localization of RNase J and RhpA by confocal fluorescence microscopy in live H. pylori cells. For this, we constructed H. pylori B128 strains with the gene encoding RNase J fused at its 3′-end to the gene encoding GFPmut2 fluorescent protein or the gene encoding RhpA fused at its 3′-end to the gene encoding supercyan fluorescent protein (SCFP). The *rnj-gfp* and *rhpA-cfp* fusion genes were introduced into the chromosome and expressed from their native promoters, replacing the original copies of *rnj* and *rhpA*, respectively (Fig. S2A). Adding a tag to the C-terminus of RNase J and RhpA does not seem to prevent their functions, as both fusion strains showed no growth defect, taking into account that RNase J is an essential gene and that the Δ*rhpA* mutant has a considerable growth defect (12, 13). The strains carrying these fusions preserved the characteristic spiral shape and had no morphological defects as visualized by phase-contrast microscopy (Fig. S2B). Western blotting performed with anti-RNase J and anti-RhpA antibodies showed that the fusion proteins were well expressed and did not undergo *in vivo* degradation (Fig. S2C).

By cellular fractionation, we found that the RNase J-GFP fusion membrane localization was similar to that of native RNase J (Fig. S2C). In contrast, we observed that the RhpA C-terminal CFP fusion causes a partial delocalization of RhpA toward the soluble extract compared to the native RhpA protein (a similar delocalization was observed with a RhpA N-terminal CFP fusion).

**FIG 2:**
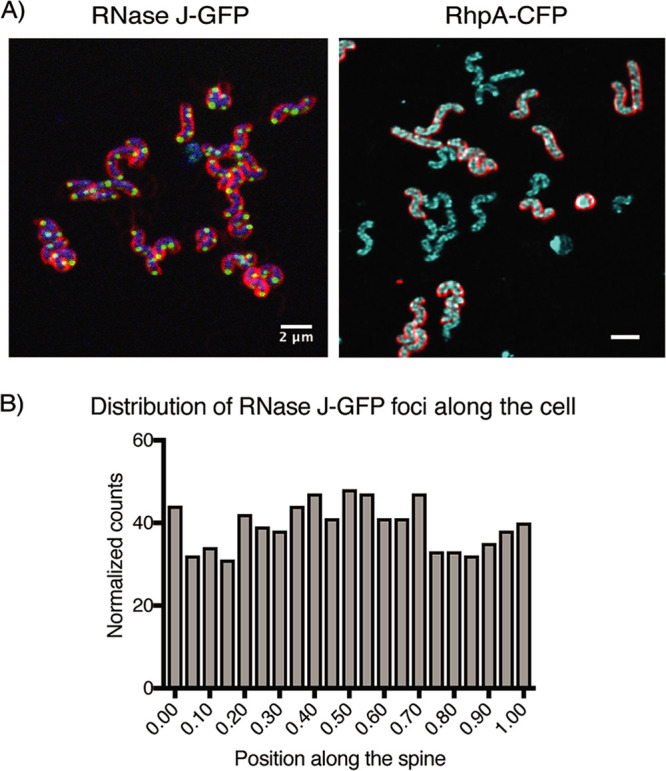
RNase J and RhpA form foci in H. pylori cells that do not have a polar localization. (A) Representative composite confocal microscopy images of live H. pylori cells expressing RNase J-GFP (green) or RhpA-CFP (cyan). In the RNase J-GFP image, blue indicates DNA (Hoechst 33342); in both images, red indicates membranes (FM4-64). Experiments were performed at least 3 times. (B) Histogram showing the number of foci of RNase J-GFP that are located in each position along the spine of the H. pylori cells.

Confocal fluorescence microscopy revealed that RNase J-GFP was visible as intense discrete foci that lay at the periphery of live bacteria (Fig. 2A). In the case of RhpA-CFP, spots were also detected at the periphery of the bacteria, although with more background throughout the cell (Fig. 2A). This was probably caused by the partial membrane delocalization of this fusion as mentioned above.

There are very few reports on GFP fusions in live H. pylori and none on CFP fusions. Therefore, several controls were performed to ensure that the degradosome foci were not generated by self-aggregation of GFPmut2 and SCFP fusions in H. pylori. H. pylori overexpressing from a plasmid either GFPmut2 or SCFP alone displayed no foci but rather an intense diffuse fluorescence signal distributed all over the cells (Fig. S2D). In addition, immunofluorescence using anti-FLAG antibodies on fixed H. pylori cells expressing RNase J-FLAG or RhpA-FLAG fusions from the chromosome revealed foci similar to those of live cells (Fig. S2E).

We then analyzed whether the RNase J-GFP foci localized to a preferential site in the cell, such as the poles. As a first step to measure the amount of polar foci, the nucleoid signal was used to assign each detected focus to the corresponding parental cell. Then, a central axis for the cells was established and the foci were assigned to the point nearest to them within the axis, in such a way that if a focus were assigned to the extremes of the axis, it would be classified as a polar focus whereas if it were assigned to the middle of the axis, it would be classified as septal. With a total of 1,403 foci analyzed, we found that they were randomly distributed in the cells and that only 5% of the total foci were located at the poles (Fig. 2B).

The foci formed by the RNase E degradosomes, either membrane associated as in E. coli or cytoplasmic as in C. crescentus, were reported to be dynamically assembled (1). Thus, the position of the RNase J-associated degradosome foci was monitored in live H. pylori cells every 6 s during 3 min and compared with those of fixed cells. No significant movement of the foci was detected, suggesting that the RNase J-based foci are static in H. pylori (Fig. S2F; see also Videos S1 and S2 in the supplemental material).

10.1128/mBio.01173-20.5 VIDEO S1 Time-lapse of live H. pylori expressing RNase J-GFP. The overlay of the phase-contrast and GFP channels is shown. Download Video S1, MOV file, 4.2 MB. Copyright © 2020 Tejada-Arranz et al. 2020 Tejada-Arranz et al. This content is distributed under the terms of the Creative Commons Attribution 4.0 International license.

10.1128/mBio.01173-20.6 VIDEO S2 Time-lapse of fixed H. pylori expressing RNase J-GFP. The overlay of the phase-contrast and GFP channels is shown. Download Video S2, MOV file, 3.5 MB. Copyright © 2020 Tejada-Arranz et al. 2020 Tejada-Arranz et al. This content is distributed under the terms of the Creative Commons Attribution 4.0 International license.

Our data show that the proteins of the H. pylori degradosome form foci that are likely physiologically compartmentalized structures. Analysis of the RNase-J foci revealed that they are (i) static and (ii) nonpolar but rather randomly distributed along the cell.

### Growth phase and antibiotic treatment impact the formation of RNase J-GFP foci.

**FIG 3:**
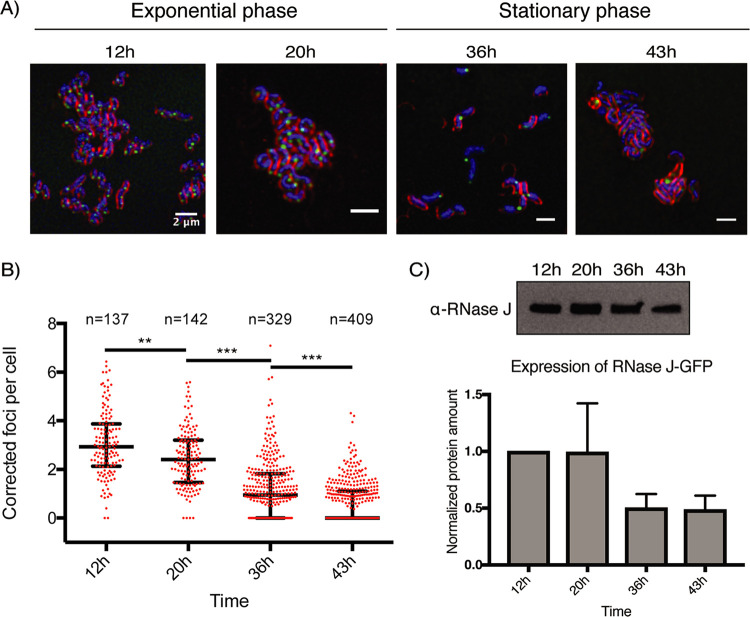
The number of RNase J-GFP foci per cell is progressively reduced along the H. pylori growth curve. (A) Representative composite confocal microscopy images of the RNase J-GFP-expressing strain at different time points (in hours) along the growth curve. Blue, DNA (labeled with Hoechst 33342); green, RNase J-GFP; red, the membrane (labeled with FM4-64). The experiment was performed in triplicate. (B) Quantification of the amount of foci per cell, normalized by the nucleoid area, along the growth curve. “n” corresponds to the number of cells analyzed for each condition. The median value is represented by a horizontal bar, and the error bars correspond to the interquartile range. *, *P* < 0.05; **, *P* < 0.005; ***, *P* < 0.0005. (C) Western blot (upper panel) and quantification (lower panel) of RNase J-GFP of cells taken at different time points along the growth curve and normalized on total proteins. The experiments were reproduced twice. The differences are not statistically significant (*P* value of 0.11).

As we showed that the RNase J-GFP fusion protein localizes like the native RNase J, we decided to use this fusion to further investigate what factors might influence focus formation. First, we quantified the number of foci per cell by automatically detecting the focus particles and assigning them to the nearest nucleoid. Every measure was normalized to the area of each nucleoid in order to avoid an overestimation of the number of foci due to dividing cells. The median number of foci per nucleoid was about 3 during the exponential-growth phase, with a range between 0 and 6 (Fig. 3AB).

Next, we wanted to check whether there was an effect of the growth phase on the amount of foci per cell. At the time point at which we took H. pylori bacteria in stationary phase, all cells had a spiral shape. We observed that the median number of detected RNase J-GFP foci steadily reduced throughout the growth phase, with medians of 2.4 foci/cell at 20 h (exponential-phase cells), 0.95 foci/cell at 36 h, and 0 foci/cell at 43 h (stationary-phase cells) (Fig. 3AB). Cell fractionation and Western blotting using anti-RNase J antibodies indicated that the levels of RNase J-GFP were indeed slightly reduced in stationary phase but not sufficiently to explain the disappearance of the foci (Fig. 3C).

**FIG 4:**
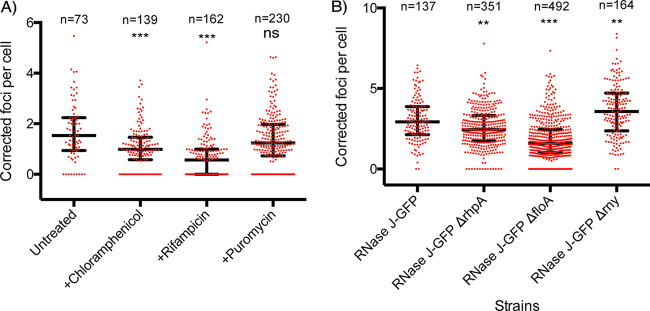
The number of RNase J-GFP foci per cell is affected by antibiotics and in different mutants. “n” corresponds to the number of cells analyzed for each condition. The median value is represented by a horizontal bar, and the error bars correspond to the interquartile range. Experiments were performed in triplicate. *, *P* < 0.05; **, *P* < 0.005; ***, *P* < 0.0005; “ns,” nonsignificant. (A) Quantification of the number of RNase J-GFP foci, normalized by the nucleoid area, in untreated cells and upon treatment with chloramphenicol, rifampicin, or puromycin. (B) Quantification of the number of RNase J-GFP foci, normalized by the nucleoid area, in wild-type cells and in bacteria deleted for the genes encoding RNase Y, flotillin, or RhpA.

In eukaryotes, p-bodies and stress granules require untranslated mRNAs to assemble. Similarly, formation of the RNase E cytoplasmic foci (BR bodies) in C. crescentus relies on free mRNAs (22). Therefore, we tested whether 30-min treatments with antibiotics known to affect the amount of translating or ribosome-free mRNAs in cells would alter the number of foci in exponentially growing H. pylori bacteria. No effect on nucleoid condensation was observed under these conditions. The antibiotics used were rifampicin, which inhibits transcription and, as a consequence, causes a decrease in the total amount of mRNAs, and chloramphenicol, which blocks ribosomes on the mRNAs that they are translating, reducing the pool of ribosome-free mRNAs. Upon treatment with rifampicin and chloramphenicol, we found a significant decrease in the median amount of foci per cell that was more pronounced in the case of rifampicin (63% reduction) than with chloramphenicol (36% reduction) (Fig. 4A) compared with untreated cells. Finally, no significant differences in the number of foci were observed after treatment with puromycin (Fig. 4A), an antibiotic whose use leads to dissociation of the ribosomal subunits, increasing the amounts of untranslated cytoplasmic mRNAs.

Furthermore, we examined whether the membrane localization of RNase J was perturbed by either the growth phase or the antibiotic treatments and we found that RNase J remained associated with the inner membrane (Fig. S3AB).

10.1128/mBio.01173-20.3 FIG S3 The cellular distribution of RNase J-GFP is not affected during growth or upon antibiotic treatment or in Δ*rny*, Δ*floA*, or Δ*rhpA* mutants. (A) Western blot with antibodies against RNase J on samples subjected to fractionation at different time points along the growth curve. (B) Western blot with antibodies against RNase J on samples subjected to rifampicin (Rif), chloramphenicol (Cam), or puromycin (Puro) treatment and fractionation. (C) Western blot with antibodies against RNase J on samples from different mutants in exponential phase that were subjected to fractionation. TE, total extract fraction; SE, soluble extract fraction; IM, inner membrane fraction. Download FIG S3, TIF file, 1.5 MB. Copyright © 2020 Tejada-Arranz et al. 2020 Tejada-Arranz et al. This content is distributed under the terms of the Creative Commons Attribution 4.0 International license.

These data suggest that ribosome-free mRNAs are among the factors promoting focus formation in H. pylori and that the formation of foci is not solely correlated with the amount of RNase J-GFP.

### Flotillin and RNase Y influence the number of RNase J-GFP foci.

As shown above, RhpA, RNase Y, and flotillin did not significantly alter the membrane localization of RNase J in H. pylori. However, we wondered whether these proteins might impact the formation of foci. Therefore, Δ*rny*, Δ*floA* and Δ*rhpA* mutations were introduced into the strain expressing RNase J-GFP and the number of foci per cell was quantified in exponential phase. We verified that the membrane localization of the RNase J-GFP fusion was not modified in these mutants (Fig. S3C) and that the nonpolar distribution of the foci was conserved.

We found that the median number of foci was significantly increased (by 22%) in the Δ*rny* strain (Fig. 4B). In both the Δ*rhpA* and Δ*floA* mutants, the amount of foci was found to be reduced (by 17% and 46%, respectively), with the effect being much stronger in the flotillin-deficient mutant (Fig. 4B). Taken together, these results indicate that the number of foci per cell is not linearly correlated with the amounts of RNase J-GFP protein and that additional factors, such as the growth phase, flotillin, and RNase Y, determine their formation.

### Superresolution microscopy to quantify the RNase J-based degradosome foci.

We decided to explore in greater detail the properties of the RNase J degradosome foci in H. pylori in both the exponential and stationary phases, for which we applied dSTORM (direct stochastic optical reconstruction microscopy), a powerful tool that allows determination of the location of individual molecules in the *x*, *y*, and *z* axes with 15-nm precision. For this analysis, H. pylori was fixed and permeabilized, membranes were labeled with wheat germ agglutinin (WGA) lectin coupled to Alexa Fluor 555 (WGA-AF555), and GFP was labeled with anti-GFP nanobodies coupled to sulfocyanin 5 (GFP-Cy5), allowing us to analyze the localization of RNase J-GFP at the single-molecule level.

**FIG 5:**
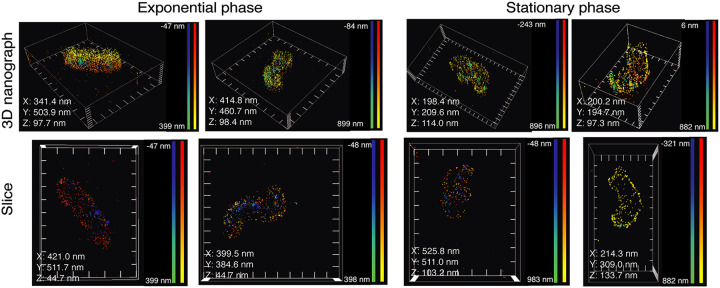
Visualization of the foci of RNase J-GFP in exponential and stationary phase by dSTORM superresolution microscopy. Representative single-molecule localization microscopy images show the membrane of H. pylori as visualized after labeling with WGA-AF555 (yellow-red) and the RNase J-GFP foci after labeling with anti-GFP-Cy5 nanobodies (blue-green). The upper panels show the full 3D volume of representative cells, and the lower panels show a 2D longitudinal slice of the bacteria. The X, Y, and Z values in each image indicate the distance between the ticks of the respective axes in the picture. The color gradients red-yellow (for WGA-AF555) and blue-green (for anti-GFP-Cy5 nanobodies) indicate the distance for each dot with respect to the coverslip, as indicated in the corresponding scale bars in each panel. The experiment was performed 3 times.

**FIG 6:**
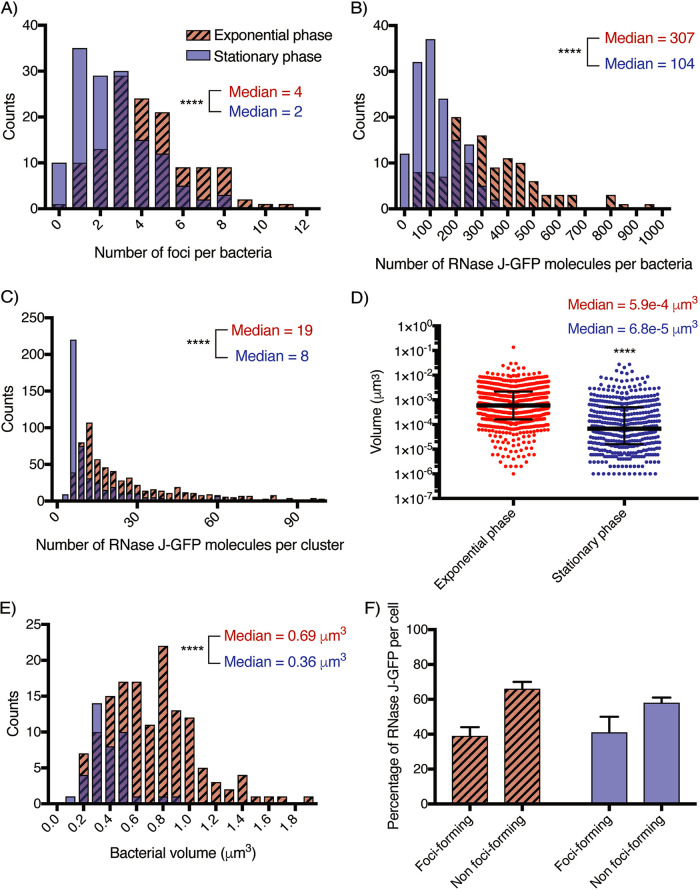
dSTORM quantification of the RNase J-GFP foci of H. pylori cells in exponential phase (red hatched bars) and stationary phase (blue bars). The experiment was carried out 3 times, and the data correspond to the results presented in Fig. 5. (A) Distribution histogram of the amount of foci per cell in exponential-phase cells (*n* = 121 cells) and stationary-phase cells (*n* = 191 cells). Median values determined under both conditions are indicated. (B) Distribution histogram of the number of RNase J-GFP molecules per cell in exponential-phase and stationary-phase cells. Median values determined under both conditions are indicated. (C) Distribution histogram of the number of RNase J-GFP molecules per cluster in exponential-phase and stationary-phase cells (*n* = 535 clusters in exponential phase and *n* = 371 clusters in stationary phase). Median values determined under both conditions are indicated. (D) Distribution histogram of the volume of the RNase J-GFP clusters in exponential-phase and stationary-phase cells. Median values determined under both conditions are indicated. (E) Distribution histogram of the volume of H. pylori cells in exponential phase (*n* = 142 cells) and stationary phase (*n* = 40 cells). ****, *P* < 0.0001 (for panels A to E). (F) Bar graph of the proportions of focus-forming and non-focus-forming RNase J-GFP molecules calculated on the basis of mean values in exponential-phase and stationary-phase cells.

First, we observed that the foci of RNase J-GFP were indeed located at the membrane in both exponential phase and stationary phase (Fig. 5; see also Videos S3 to S6). In addition, the higher sensitivity of superresolution microscopy allowed us to visualize more foci than conventional confocal microscopy. A median number of 4 foci per cell was observed in exponential phase and of 2 in stationary phase (Fig. 6A), in agreement with the reduction in the number of foci seen by confocal microscopy. Furthermore, we were able to quantify the number of RNase J-GFP molecules per focus and analyze their dimensions in both phases. In exponential phase, we measured a median of 307 RNase J-GFP molecules per cell (Fig. 6B), with a median of 19 RNase J-GFP molecules in each focus (Fig. 6C) that presented a median volume of 5.91e−4 μm^3^ (Fig. 6D). In stationary phase, we detected a median of 104 total molecules per cell (Fig. 6B), and the foci contained a median of 8 molecules (Fig. 6C) and presented a volume of 6.75e−5 μm^3^ (Fig. 6D), approximately nine times less than the foci in exponential phase. Therefore, the foci were smaller and contained fewer RNase J-GFP molecules in stationary phase. As expected, the cell volume also changed during growth; a median volume of 0.7 μm^3^ was measured in exponential phase and changed to 0.36 μm^3^ in stationary phase (Fig. 6E). If we correct by the bacterial volume, the concentration of RNase J molecules per cell was marginally changing between the exponential and stationary phases and so was the number of foci per cell, whereas the volume of foci and the amount of RNase J per foci suffered more important changes. Therefore, we can conclude that the amount and size of the RNase J foci are scaled with the cell volume and thus with the growth stage.

Finally, we also found that in both growth phases, the mean proportions of total RNase J-GFP molecules that were clustered in foci were the same (40%), irrespective of the total amount of RNase J-GFP (Fig. 6F). Thus, the foci are not the consequence of RNase J molecules reaching a threshold concentration that causes them to spontaneously aggregate into foci but are instead formed by a regulated mechanism as suggested above. Furthermore, the fact that a significant (60%) fraction of RNase J-GFP molecules did not form foci (Fig. 6F) suggests the existence of two different populations of RNase J that may play different physiological roles.

## DISCUSSION

In this work, we uncovered the inner membrane association of the H. pylori RNA degradosome. RNase J and RhpA, the two protein partners of this minimal degradosome, present no predictable membrane targeting sequences; they interact with the inner membrane through hydrophobic and electrostatic interactions and are thus peripheral membrane proteins of H. pylori. This membrane localization of the RNA degradosome is independent of RNA and relies neither on the membrane-bound RNase Y nor on the sole flotillin of H. pylori. The association of the degradosome partners with the H. pylori membrane might, however, be mediated by another protein, yet to be identified. During our previous studies, RNase J and RhpA were detected in association with purified ribosomes (70S particles and polysomes) (11). The present results show that the membrane localization of the degradosome is not mediated by or dependent on ribosomes. Thus, two different possibilities arise: (i) the ribosome-associated degradosome fraction corresponds to the small amount of proteins detected in the soluble fraction, which might have a function different from that of the membrane-bound population, or (ii) the small ribosome-bound fraction of the degradosome is also attached to the membrane and plays a role only with ribosomes that are localized in the vicinity of the membrane.

Only a few previous studies have reported the subcellular localization of RNA degradosomes in other bacteria. The localization varied depending on the organism and did not correlate with the nature of the scaffolding RNase that it contains. In E. coli, the degradosome is targeted to the inner membrane through an amphipathic helix of RNase E, and deletion of this membrane anchor results in a global slowdown of RNA degradation (31). Strikingly, this degradosome also presents both inner membrane and ribosomal localizations (20, 32). We believe that this “evolutionary convergence” indicates that the dual localizations of the RNA degradosome might have a functional significance. In contrast, RNase E of C. crescentus does not contain this helix and is cytoplasmic (21). Concerning the potential RNase Y-based degradosome of the Gram-positive bacterium B. subtilis, the results were rather contradictory. RNase Y is, as expected, membrane associated, but RNase J1, which is one of its proposed interacting partners, does not follow the same distribution, being cytoplasmic (25, 33). In contrast, in a recent work, a fraction of RNase J2 of Streptococcus mutans was found to localize at the membrane (34). In B. subtilis, RNase Y was recently found to assemble at the membrane into dynamic short-lived foci that increased in number and size upon rifampicin treatment or deletion of the Y-complex, a protein complex that modulates RNase Y activity (27). Those data suggest that RNase Y foci represent a less active form of the enzyme in contrast to the situation of RNase E foci (27).

The differences in the localizations of bacterial degradosomes might also be related to the diversity in the repertoires of degradosome RNases observed in many bacteria (1). It is most probable that different types of degradosomes with different localizations coexist in numerous bacteria, pointing to interesting issues regarding their respective functions, potential cross talk, and target specificities.

Fluorescent fusions of both partners of the RNA degradosome were analyzed in live H. pylori cells by confocal microscopy. RNase J-GFP formed bright foci at the cell periphery that were nonpolar. The results seen with the RNase J foci, shown to be present at a median of 3 per bacterium in exponential phase by confocal microscopy, suggest that RNA degradosome complexes are located at discrete sites at the cell membrane. Under the same conditions, RhpA-CFP also formed foci at the periphery of the cell. Although we favor the hypothesis of “mixed” RNase J-RhpA degradosome foci, this could not be established since colocalization studies were prevented by the presence of fluorescence background throughout the RhpA-CFP-expressing cells, which we could attribute to partial membrane delocalization of the RhpA-CFP fusion.

The RNase J-GFP fusion was further used to identify factors that modify its cellular distribution. We identified several factors important for focus formation, none of which significantly impacted the membrane localization of RNase J and of RhpA. We found that RNase J focus formation was impacted by several mutations (Δ*rhpA*, Δ*rny*, Δ*floA*). The number of RNase J foci per cell was slightly diminished in the absence of its RhpA partner. In the absence of RNase Y, the number of foci per cell increased by 22%. One plausible explanation, supported by the antibiotic treatment data, is that focus formation depends on the amount of free RNA and would, in the mutant deficient in RNase Y, be promoted by the accumulation of its RNA targets. In the absence of the sole flotillin of H. pylori, the number of foci per cell was reduced by 46%, showing that this membrane scaffolding protein promotes focus formation. In S. aureus, FloA affects the function and oligomeric state of the membrane-bound RNase Y (28). The H. pylori *ΔfloA* mutant presents no growth defect, suggesting that RNase J activity is not significantly affected. However, FloA-rich membrane regions might act as scaffolds to bring molecules together, maybe fostering oligomerization and focus formation.

We examined how the exposure of H. pylori cells to three different antibiotics impacted the formation of RNase J-GFP foci. These treatments did not change the membrane targeting of RNase J-GFP. Rifampicin, a transcription inhibitor, and chloramphenicol, a translation inhibitor that locks ribosomes on mRNAs, are known to lower the intracellular concentration of untranslated/free RNAs. These two antibiotics cause a significant reduction in the number of foci per cell (the effect being more pronounced with rifampicin). This suggests that free/untranslated RNA is a factor promoting RNase J focus formation and that the number of degradosome foci is probably adjusted to the amount of free cellular RNAs, their proposed targets. Treatment with puromycin, an antibiotic whose presence leads to the dissociation of the ribosomal subunits, thereby increasing the amounts of untranslated free mRNAs, did not significantly change the number of foci per cell. Our interpretation is that, under this condition, the changes in the free RNA concentration are not sufficient to significantly impact focus formation. Altogether, our results show that RNA does not determine the membrane localization of RNase J and instead suggest that free/untranslated RNA is a factor promoting RNase J focus formation. We interpret this as an indication that these clusters might represent the active form of the RNA degradosome. In addition, we also observed that not all RNase J proteins present at the membrane were embedded in foci, an aspect that we were able to tackle with superresolution microscopy.

In B. subtilis, rifampicin treatment does not change RNase Y membrane localization, although it modifies the number of RNase Y foci, and results in complete delocalization of RNase J1 and J2 (25, 27). In E. coli, rifampicin treatment results in the disappearance of RNase E-YFP foci and an increased rate of RNase E membrane diffusion (20). In C. crescentus, using three-dimensional (3D) single-particle tracking and superresolution microscopy, it was observed previously that the number of confined/clustered RNase E molecules decreases upon rifampicin treatment (21). Another study reported that the number of C. crescentus RNase E foci was reduced upon rifampicin, chloramphenicol, or tetracycline treatment and slightly increased upon puromycin treatment (22). Thus, untranslated RNA appears to play an important role in RNA degradosome focus formation/clustering in distantly related microorganisms (H. pylori, E. coli, and C. crescentus) and for unrelated RNases (RNase J and RNase E), pointing to evolutionarily conserved features.

Our data discussed so far suggest that the formation of RNase J foci is not constitutive. By confocal microscopy, the number of foci was found to diminish during the H. pylori growth phase, resulting in almost no detectable foci in stationary phase. However, in stationary phase, the total amount of RNase J-GFP proteins was slightly diminished whereas its membrane localization was maintained. Using dSTORM, we found the number of RNase J molecules per cell in stationary phase to be approximately half that in exponential phase, and we could detect more foci than with confocal microscopy, with median numbers of 4 and 2 foci per cell in the exponential and stationary phases, respectively. Between the exponential and stationary phases, the median volume of the foci was decreased by 9-fold and contained half the number of RNase J molecules. Thus, the volume of the foci was not linearly proportional to the number of RNase J molecules that each contained, suggesting that the composition and, possibly, the activity of foci change during growth. Importantly, the mean proportion of RNase J molecules contained by the foci remained the same (about 40%) between the exponential and stationary phases.

Our dSTORM data allowed us to quantify the cell volume of H. pylori as a function of growth, and we found, similarly to what was established in E. coli, that the volume was approximately two times lower in stationary phase. We then calculated that the RNase J “cellular concentration” and the number of foci per cell were approximately constant along the growth curve and conclude that the RNase J foci were undergoing a process of cell size scaling. This is particularly interesting since eukaryotic ribonucleoprotein granules depend on cell size for assembly (35). Such scaling processes allow the cells to adapt their functions within organelles or membraneless organelles to their volume changes during growth.

These data suggest that assembly of foci is indeed a regulated process and is not due to spontaneous assembly of proteins. Our results also suggest that free RNA contributes to focus formation, through a mechanism that remains to be elucidated. The bacterial mRNA concentration is known to be reduced in stationary phase (36) and thus might constitute one important factor controlling the evolution of foci during growth in H. pylori.

A dual pattern of behavior during the exponential and stationary phases has been proposed for RNases J1 and J2 from Streptococcus pyogenes, and it was previously suggested that these RNases might be less active during stationary phase (37). It was also previously shown that, in S. pyogenes, RNase J1 and J2 (J1/2) are necessary only for the initial step of decay of the transcripts that are degraded in stationary phase (37).

The E. coli and C. crescentus degradosomes were also found to assemble into foci or clusters (20–22). These clusters are highly dynamic, while those of H. pylori are, under the tested conditions, static. In addition, the cytoplasmic clusters of the RNase E-based C. crescentus degradosome have LLPS properties similar to those of eukaryotic messenger ribonucleoprotein (mRNP) granules (like p-bodies or stress granules). Despite their differences, the three types of clustered bacterial degradosomes reported so far (including that of H. pylori) present striking similarities to these eukaryotic structures (some of which are static [38, 39]); they all form compartmentalized structures within the cell, their formation is regulated and promoted by RNA, and for H. pylori they seem to be scaled to the size of the cells. The formation of LLPS structures is frequently linked to RNA-binding proteins and proteins with intrinsically disordered regions (IDRs) that can act as intermolecular interaction hot spots. The C-terminal domain of *Eco*RNase E is an IDR (40). Using the IUPred2A algorithm to predict IDRs (41), such a region was predicted in the N-terminal part of RNase J of H. pylori (first 100 amino acids [aa]; Fig. S1C), a region that is specific to *Helicobacter* RNase J proteins. An IDR is also predicted in the C terminus (last 60 aa) of RhpA (Fig. S1D). These predictions remain to be experimentally validated.

It is still not clear whether the bacterial degradosome foci correspond to active RNA degradation hubs or to a storage form of the enzymes and/or RNA molecules or other proteins. Surprisingly, after many years, this issue is still partially unresolved for p-bodies and stress granules. However, recent data obtained in C. crescentus suggest that they are active structures (23).

**FIG 7:**
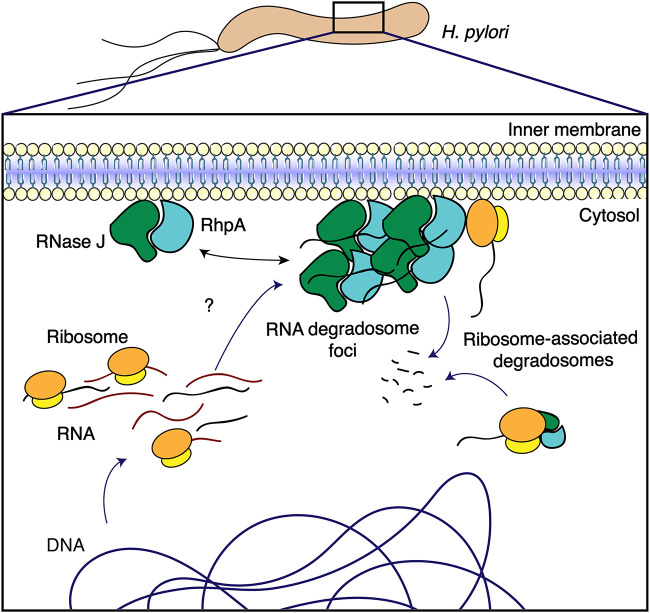
Model for the regulation of the RNA degradosome foci in H. pylori. RNase J and RhpA, the two protein components of the RNA degradosome, are associated with the H. pylori inner membrane. Minor proportions of these proteins are associated with translating ribosomes, which could be either cytoplasmic or located at the membrane. RNase J and RhpA assemble into foci at the membrane, probably together. On the basis of our data, we propose a model where foci represent the active form of the RNA degradosome, constituting RNA degradation hubs. Comparatively, the complexes located outside foci would retain little or no activity. In this model, target RNA molecules would be directed to the foci for degradation by an unknown mechanism, providing in addition a spatiotemporal delay that allows for their translation before their degradation. The RNA degradosomes associated with ribosomes could be involved either in rRNA maturation or in coupling between translation and mRNA degradation.

We propose a working model for the dual localizations of the degradosome (Fig. 7). The membrane association of the degradosome could represent a manner to compartmentalize RNA degradation, and an active form of the degradosome would be clustered into foci. This could allow posttranscriptional regulation of a subcategory of genes whose mRNAs are directed to the foci by an unknown mechanism while also providing a spatiotemporal delay to allow transcribed mRNAs to be translated before their degradation. The ribosome-associated degradosomes (be they at the membrane and forming foci or not) could be in charge of degrading RNA molecules that need to be tightly regulated and that would be deleterious if left unchecked, they could degrade defective RNA molecules or play a role in rRNA maturation, as has been shown for RhpA (13).

In conclusion, we have discovered and characterized the membrane localization and clustering of the RNA degradosome in the important pathogen H. pylori. We propose that the balance between this localization and its clustering and its ribosomal association represent major levels of control of its activity and specificity that might be more generally relevant and open many exciting perspectives of research by analogy with the equivalent eukaryotic structures.

## MATERIALS AND METHODS

### Bacterial strains and growth conditions.

The H. pylori strains used in this study (Table S1) were derivatives of strain B128 (42, 43). Plasmids (Table S1) used to create mutants of H. pylori were constructed and amplified using Escherichia coli One Shot TOP10 or DH5α strains (Thermo Fisher). H. pylori strains were grown on blood agar base 2 (Oxoid) plates supplemented with 10% defibrinated horse blood and with the following antibiotic-antifungal cocktail: amphotericin B 2.5 μg ⋅ ml^−1^, polymyxin B 0.31 μg ⋅ ml^−1^, trimethoprim 6.25 μg ⋅ ml^−1^, and vancomycin 12.5 μg ⋅ ml^−1^. Selection of H. pylori mutants was performed using kanamycin 20 μg ⋅ ml^−1^, apramycin 10 μg ⋅ ml^−1^, or chloramphenicol 10 μg ⋅ ml^−1^. For liquid cultures, we used Brucella broth supplemented with 10% fetal calf serum (FCS) (Eurobio), the antibiotic-antifungal cocktail, and the selective antibiotic when necessary. H. pylori cells were grown at 37°C under microaerophilic atmosphere conditions (6% O_2_, 10% CO_2_, 84% N_2_) using an Anoxomat (Mart Microbiology) atmosphere generator.

10.1128/mBio.01173-20.4 TABLE S1 Strains, plasmids, and oligonucleotides used in this study. Download Table S1, DOCX file, 0.03 MB. Copyright © 2020 Tejada-Arranz et al. 2020 Tejada-Arranz et al. This content is distributed under the terms of the Creative Commons Attribution 4.0 International license.

### Molecular techniques.

Molecular biology experiments were performed according to standard procedures (44) and the recommendations of the supplier (Fermentas). A NucleoBond Xtra midi kit (Macherey-Nagel) and a QIAamp DNA minikit (Qiagen) were used for plasmid preparations and H. pylori genomic DNA extractions, respectively. PCR was performed either with DreamTaq DNA polymerase (Thermo Fisher) or with Q5 High-Fidelity DNA polymerase (NEB) when the product required high-fidelity polymerase.

### Construction of H. pylori mutants and fusions.

Chromosomal deletion of the entire genes encoding RNase Y (HPB128_186g30), RhpA (HPB128_21g22), and flotillin (HPB128_21g23) was performed in strain B128. Briefly, fragments of about 500 bp upstream and downstream of the target gene were amplified by PCR (oligonucleotides are listed in Table S1) and spliced into a nonpolar kanamycin, chloramphenicol, or apramycin resistance cassette by using a Gibson isothermal assembly kit (NEB) followed by PCR amplification using the primers from the extremities. All H. pylori mutants were obtained by natural transformation (as described previously [45]) with the PCR fragments obtained as described above. Selection of chromosomal allelic exchange resulting in gene deletion was performed with the corresponding antibiotic. Deletion of the genes of interest and correct insertion of cassettes were verified by PCR and sequencing of the gene region. RNase J-GFP, RNase J-FLAG, RhpA-CFP, and L9-V5 fusions were also constructed using the isothermal assembly technique (46). Briefly, target genes and GFPmut2 (47) or V5 tag (48) followed by the kanamycin resistance cassette or target genes and SCFP (49) followed by the chloramphenicol resistance cassette or target genes and FLAG tag (50) followed by the apramycin resistance cassette were fused in that order after amplification using complementary primers (described in Fig. S2A). A linker sequence (4 aa; sequence Phe-His-Gly-Ser) was introduced between RNase J/RhpA and the fluorescent proteins in RNase J-GFP and RhpA-CFP fusions. Then, the final construction was amplified by PCR and directly introduced into H. pylori by natural transformation. Correct insertion of the fusion and cassette was verified by PCR, sequencing, Western blotting, and, when relevant, microscopy. Additionally, the *gfpmut2* (51) and *scfp* genes were cloned into the pILL2157 shuttle vector that replicates in H. pylori (52) between the NdeI and BamHI restriction sites under the control of an IPTG (isopropyl-β-d-thiogalactopyranoside)-inducible promoter. These constructs were transformed into H. pylori and verified by PCR and sequencing and served as controls for microscopy analysis.

### Confocal fluorescence microscopy.

Bacterial samples taken from cultures with different optical densities at 600 nm (OD_600_) were concentrated or diluted to an OD_600_ of 2 and, when necessary, stained. The cultures were labeled with NucBlue live-cell stain ReadyProbes reagent (Hoechst 33342; Molecular Probes) for DNA per the recommendations of the supplier and with 4 μg/ml FM4-64 (Thermo Fisher Scientific) for membranes. For strains carrying the RhpA-CFP fusion, only FM4-64 was used. After 20 min of incubation in the dark, bacterial samples were spotted on microscope slides with a thin layer of 1% agarose–phosphate-buffered saline (PBS). The agarose pad was covered by a coverslip, and samples were directly observed at room temperature. Images of strains expressing RNase J-GFPmut2 or RhpA-SCFP from the chromosome or GFPmut2 and SCFP from pILL2157 were obtained using a Leica confocal SP8 inverted microscope through a numerical-aperture (NA) 1.4 oil immersion 63× lens objective.

The detection was performed by the use of a photomultiplier tube (PMT) detector for the NucBlue channel and HyD for the other fluorophores. Images were analyzed using Fiji (53). Pictures were deconvolved using the classic maximum likelihood algorithm of Huygens software (SVI, Laapersveld, The Netherlands).

The strains expressing RNase J-FLAG and RhpA-FLAG were fixed and permeabilized with 100% methanol at −20°C for 5 min. Subsequently, the bacteria were washed with PBS and deposited on poly-l-lysine-covered coverslips. The samples were quenched for 30 min at room temperature with 100 mM glycine and were then blocked with 5% bovine serum albumin (BSA)–PBS for 1h at room temperature. They were then incubated with anti-FLAG antibody (Sigma) (1:200)–2% BSA–PBS, washed three times with 2% BSA–PBS, incubated with anti-mouse AF488 secondary antibody (Bethyl) (1:200), washed six times with 2% BSA–PBS, and mounted on Fluoromount-G (Thermo Fisher Scientific). No signal was seen when the protocol was carried out on a strain lacking a FLAG tag. Imaging and analysis were performed as described above.

### Quantification of foci.

To quantify the RNase J-GFP foci in H. pylori, images of cells were first segmented manually using the nucleoid signal, which was homogeneous. Only those cells that could be segmented with certainty were taken. RNase J-GFP foci were detected using the blob detector function with scale space (variable target blob size) from scikit-image (54). The particle size range was set to 1 to 2 pixels (corresponding to about 60 to 120 nm); the sensitivity of the blob detector was set to 0.006. This sensitivity was kept constant during analysis of all images and adjusted only in a few noisy images to suppress apparent false positives from the background.

To correct for lateral shifts (in the *xy* plane) due to chromatic aberrations, we used a standard approach utilizing fiducial markers. Multicolor fluorescent beads (TetraSpeck; Thermo Fisher) (0.2-μm-diameter microspheres) were dispersed in buffer, immobilized on a glass coverslip, and imaged in the DAPI (4′,6-diamidino-2-phenylindole) and GFP channels. Beads were detected in the images, and their *xy* positions were found with subpixel resolution using the FIJI plugin TrackMate (55). For each bead, the lateral shift (localization discrepancy between the channels) was calculated; the average shift per bead gives the single shift vector between the GFP and DAPI channels. This vector was used in the subsequent analysis scripts to obtain corrected degradosome positions (GFP channel) relative to the nucleoid positions (DAPI channels). Next, for each RNase J focus, we searched for its nearest nucleoid within a fixed search radius (twice the average nucleoid size). The distance between a nucleoid and a RNase J focus was calculated from the RNase J focus position (its center) and a nucleoid centroid. This step typically yields several nucleoid candidates, only one of which is selected, that being the one whose edge comes the closest to the focus. This allows determination of the number of degradosome foci per nucleoid.

The position of the RNase J foci with respect to the bacterial poles was determined as follows. We defined the medial axis of the nucleoid (spine) using the skeletonization function in the Python skimage library and then assigned the values 0 and 1 to either end of the spine (poles). For each RNase J focus, the closest point on the spine of its host nucleoid was found. The length of the spine between this point and the closest pole was divided by the total length of the spine to find the relative position (RP) of the RNase J focus along the spine. In this way, if for an RNase J focus the RP value is 0 or 1, it is then located at one of the poles, whereas if the RP is 0.5, it is then located at the middle of the spine. Finally, the frequencies of foci with RP = 0 or 1 were normalized by the perimeter of the pole tip to compensate for the accumulation of foci due to their rotational freedom around the tip. The code data are available at the following repository: https://gitlab.pasteur.fr/iah-public/hpyloridegradosomeanalysis.

### Mobility of foci.

To quantitatively compare the mobilities of the foci in live and fixed cells, we used mean square displacement (MSD) data as metrics. For this, foci were first tracked with subpixel resolution using the TrackMate plugin in FIJI (55). The resulting tracks were analyzed using MSDanalyzer (56) in MATLAB. The trajectories were corrected for drift using velocity correlation in MSDanalyzer.

### Fractionation of H. pylori.

The cellular fractionation protocol was adapted from a previous study (29). H. pylori cells were grown to an appropriate OD_600_ (0.7 to 1.5 for exponential phase or >3 for stationary phase) and then harvested by centrifugation and washed with PBS prior to being resuspended (normalizing to an OD_600_ of 5). Then, bacteria were disrupted by sonication in a lysis buffer containing 10 mM Tris-HCl (pH 7.4) (buffer A) and Complete protease inhibitor cocktail (Roche). Cell debris was removed by centrifugation at 16,000 × *g* at 4°C for 10 min, and supernatants were collected as total extracts. The supernatants were transferred to 1.5-ml ultracentrifugation tubes (Polyallomer; Beckman Coulter) and then centrifuged 45 min at 100,000 × *g* at 4°C in a TLA-100 ultracentrifuge (Beckman Coulter). The supernatant contained the soluble fraction, and the pellet corresponded to the total membranes. The pellet was washed once with buffer A and then resuspended in 10 mM Tris-HCl (pH 7.5)–0.1% N-lauroyl-sarcosin (Sigma-Aldrich)–Complete protease inhibitor cocktail (buffer B). After another ultracentrifugation performed under the same conditions, the supernatant contained the inner membrane and the pellet the outer membrane, which was resuspended in 10 mM Tris-HCl (pH 7.5)–1% N-lauroyl-sarcosin–Complete protease inhibitor cocktail (buffer C). At each step, each sample was suspended in the same volume to obtain samples from the same number of bacteria.

To determine the nature of the interaction of RhpA and RNase J with the inner membrane of H. pylori, we tested different treatments as described previously (57). Peripheral membrane proteins dissociate from the membrane when treated with a polar reagent that does not disrupt the lipid bilayer (urea 6 M) or exposed to extreme alkaline pH (Na_2_CO_3_ 100 mM, pH 11) or to a high salt concentration (NaCl 2 M). After ultracentrifugation for 45 min at 100,000 × *g*, peripheral proteins were found in the supernatant whereas integral membrane proteins were found in the pellet. Finally, treatments with 1 μg/ml RNase A (Thermo Fisher) or 20 mM EDTA (Sigma-Aldrich) were performed by adding them to the cells just after sonication, and those conditions were maintained during the first ultracentrifugation.

### Antibiotic treatments.

To examine the effect of antibiotics on the subcellular localization of RNase J and RhpA and the RNase J-GFP focus formation, rifampicin, chloramphenicol, or puromycin was added at 100 μg/ml, 200 μg/ml, or 150 μg/ml, respectively, to cultures in late exponential phase, as described previously (22). Cultures were further incubated for 30 min in a microaerobic atmosphere at 37°C with agitation. The cultures were then collected and treated for microscopy and/or fractionation.

### Western blotting.

Proteins were loaded and separated on a 4% to 20% Mini-Protean TGX stain-free precast protein gel (Bio-Rad) and subsequently electrotransferred to a polyvinylidene difluoride (PVDF) membrane (Bio-Rad) with a TransBlot Turbo system (Bio-Rad). The H. pylori RhpA, RNase J, AmiE, MotB, and BabA proteins were detected with rabbit polyclonal antibodies anti-RhpA, anti-RNase J (11), anti-AmiE (58), anti-MotB (gift of N. Buddelmeijer), and anti-BabA (59) at the respective dilutions of 1:5,000, 1:500, 1:500, 1:500, and 1:10,000. Goat anti-rabbit IgG-horseradish peroxidase (IgG-HRP; Santa Cruz) was used as secondary antibody at 1:10,000 dilution, and the detection was achieved with ECL Femto reagent (Thermo Fisher). The V5 tag was detected with an anti-V5 antibody coupled with HRP (Santa Cruz) (1:5,000).

### Sample preparation for fluorescence nanoscopy using single-molecule localization microscopy (SMLM).

Bacterial suspensions of the RNase J-GFP-expressing strain in exponential-growth phase (after 18 h of culture) or in stationary phase (after 40 h of culture) were fixed in 1% paraformaldehyde (Sigma)–PBS for 5 min at room temperature. Following three washes in PBS, bacteria were permeabilized using 0.05% Triton X-100–PBS. Anti-GFP nanobodies coupled with Cy5 (Fluotag-Q; NanoTag Biotechnologies GmbH) were then added (1:250) to the bacterial suspensions and incubated for 1 h at 37°C. The cells were then washed and treated with 2 μg/ml WGA-AF555 (Thermo Fisher Scientific)–PBS. In order to (i) immobilize the bacterial cells onto the coverslips (no. 1.5H; Marienfeld, Germany) and (ii) induce the photoswitching of Cy5, the bacteria were seeded onto STORM buffer-based pads (Blinking Pad kit; Abbelight, Paris, France).

### Single-molecule localization microscopy imaging and analysis.

2D and 3D images were taken using an inverted bright-field Olympus IX83 microscope equipped with an oil immersion 100× lens objective with a high numerical aperture value (1.49). To perform fluorescence nanoscopy experiments, a SAFe360 module (Abbelight, France) was added to the camera port of the microscope. This detection module couples single-molecule localization microscopy (SMLM), supercritical angle fluorescence (SAF), and astigmatism (60, 61) in a dual-view setup coupled with scientific complementary metal oxide semiconductor (sCMOS) cameras (Orcaflash v4; Hamamatsu). Prior to each acquisition, bright-light and diffraction-limited images were acquired. Using continuous excitation at 639 nm in the HiLo mode, most of the Cy5 molecules were induced into a dark state until a sufficient density was obtained (typically 1 to 5 molecules per bacterium per frame). Image series (5,000 frames) were recorded with a 50-ms exposure time. Raw images and the resulting coordinate tables were processed and analyzed using NEO SAFe software (Abbelight, France). Analysis of high-density regions of localizations was performed using the density-based clustering algorithm DBSCAN (62), a value of 75 nm for the distance parameter, and a parameter value of 5 for the minimum number of points.

The volume of H. pylori cells was calculated using the dSTORM images on the basis of the *xyz* coordinates of the detections of the membrane marker (WGA-AF555). Clusters of detections belonging to a bacterium were identified with the *XY* coordinates, and the clusters were refined by filtering by the number of points in a cluster and the radius of gyration. Each cluster was converted into a 2D polygon using the Python alphashape library, and the 2D area (*A*) of the polygon was calculated from the *xy* coordinates of the underlying cluster. Using this area, the volume (*V*) was calculated using the expression *V* = H*A, where *H* is the height of the bacteria. The height was estimated from the span of coordinates in the *z* dimension of the clusters (600 nm). The volume of the bacteria might be slightly overestimated due to the localization errors in imaging; however, this should not influence the ratio of exponential-growth-phase cells to stationary-growth-phase cells.

### Statistical analysis.

To compare the numbers of degradosome foci per cell between the different conditions, we used the nonparametric Mood’s median test, and *P* values lower than 0.05 were considered significant. The differences between the median values derived from superresolution microscopy were analyzed with the Mann-Whitney test, and *P* values lower than 0.05 were considered significant.

10.1128/mBio.01173-20.7 VIDEO S3 3D visualization of superresolution imaging data corresponding to an example of representative bacteria in exponential growth phase. The yellow-red gradient is the membrane, labeled with WGA-AF555, according to the distance to the coverslip, and the blue-green gradient is RNase J-GFP, labeled with anti-GFP-Cy5 nanobodies, according to the distance to the coverslip. Download Video S3, MOV file, 2.2 MB. Copyright © 2020 Tejada-Arranz et al. 2020 Tejada-Arranz et al. This content is distributed under the terms of the Creative Commons Attribution 4.0 International license.

10.1128/mBio.01173-20.8 VIDEO S4 3D visualization of superresolution imaging data corresponding to a second example of representative bacteria in exponential growth phase. The yellow-red gradient is the membrane, labeled with WGA-AF555, according to the distance to the coverslip, and the blue-green gradient is RNase J-GFP, labeled with anti-GFP-Cy5 nanobodies, according to the distance to the coverslip. Download Video S4, MOV file, 2.2 MB. Copyright © 2020 Tejada-Arranz et al. 2020 Tejada-Arranz et al. This content is distributed under the terms of the Creative Commons Attribution 4.0 International license.

10.1128/mBio.01173-20.9 VIDEO S5 3D visualization of superresolution imaging data corresponding to an example of representative bacteria in stationary growth phase. The yellow-red gradient is the membrane, labeled with WGA-AF555, according to the distance to the coverslip, and the blue-green gradient is RNase J-GFP, labeled with anti-GFP-Cy5 nanobodies, according to the distance to the coverslip. Download Video S5, MOV file, 1.9 MB. Copyright © 2020 Tejada-Arranz et al. 2020 Tejada-Arranz et al. This content is distributed under the terms of the Creative Commons Attribution 4.0 International license.

10.1128/mBio.01173-20.10 VIDEO S6 3D visualization of superresolution imaging data corresponding to another example of representative bacteria in stationary growth phase. The yellow-red gradient is the membrane, labeled with WGA-AF555, according to the distance to the coverslip, and the blue-green gradient is RNase J-GFP, labeled with anti-GFP-Cy5 nanobodies, according to the distance to the coverslip. Download Video S6, MOV file, 2.4 MB. Copyright © 2020 Tejada-Arranz et al. 2020 Tejada-Arranz et al. This content is distributed under the terms of the Creative Commons Attribution 4.0 International license.
