# Supplementary figures and images for: The RNase J-Based RNA Degradosome Is Compartmentalized in the Gastric Pathogen Helicobacter pylori
Source: mBio. 2020 Sep 15;11(5):e01173-20. doi: 10.1128/mBio.01173-20 (PMC7492731; doi:10.1128/mBio.01173-20)

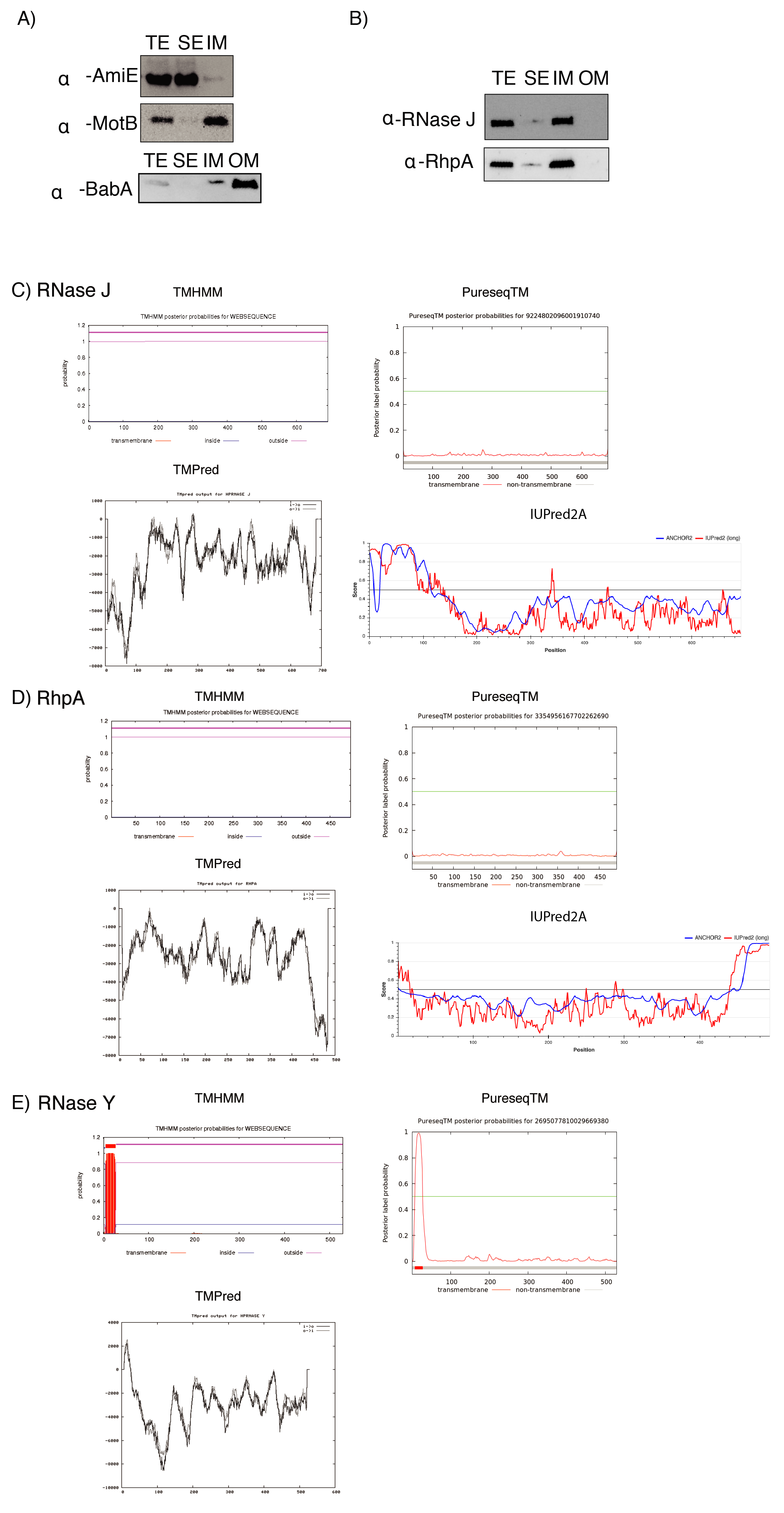

Supplement: FIG S1 [file mBio.01173-20-sf001.tif]

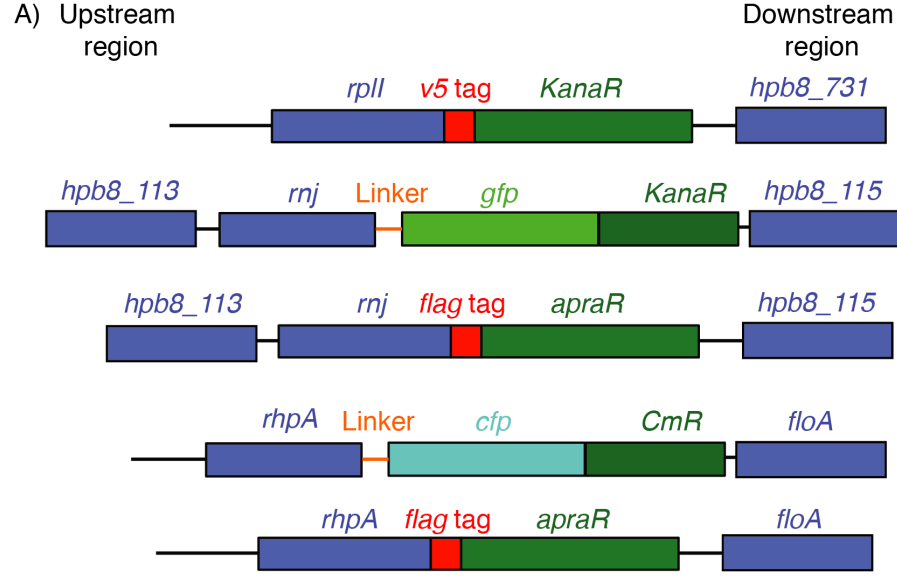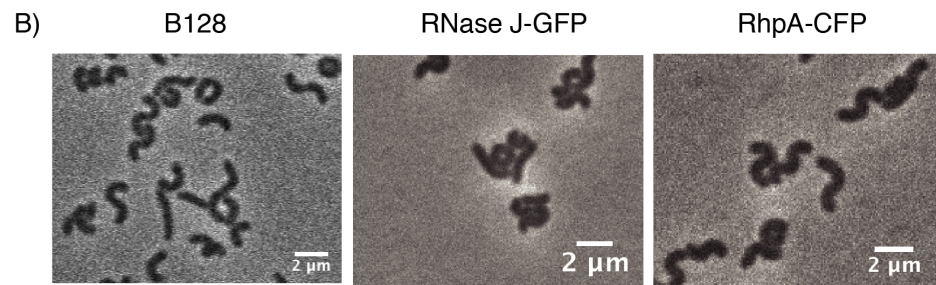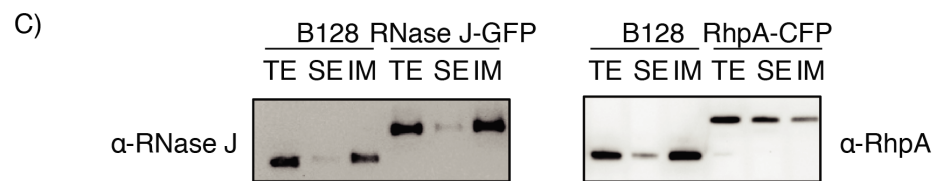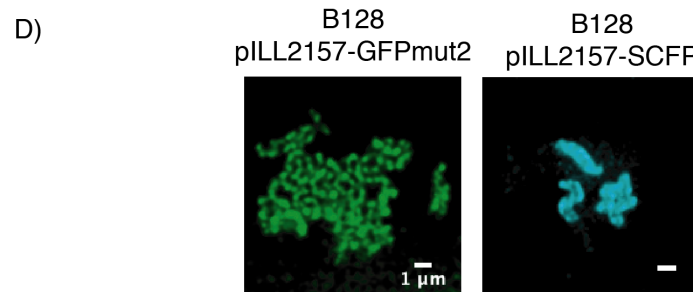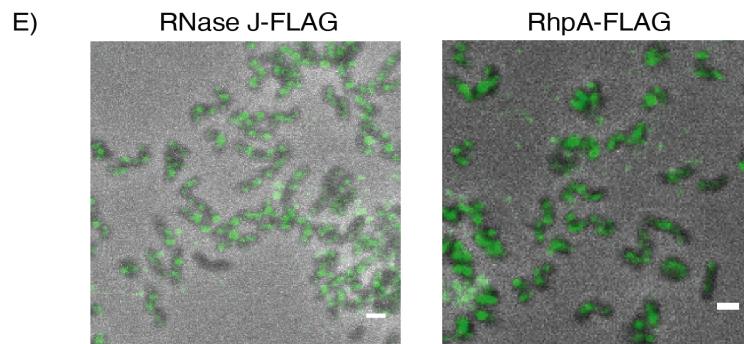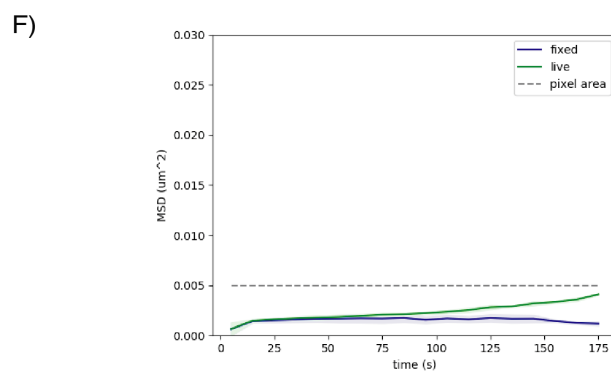

Supplement: FIG S2 [file mBio.01173-20-sf002.pdf]

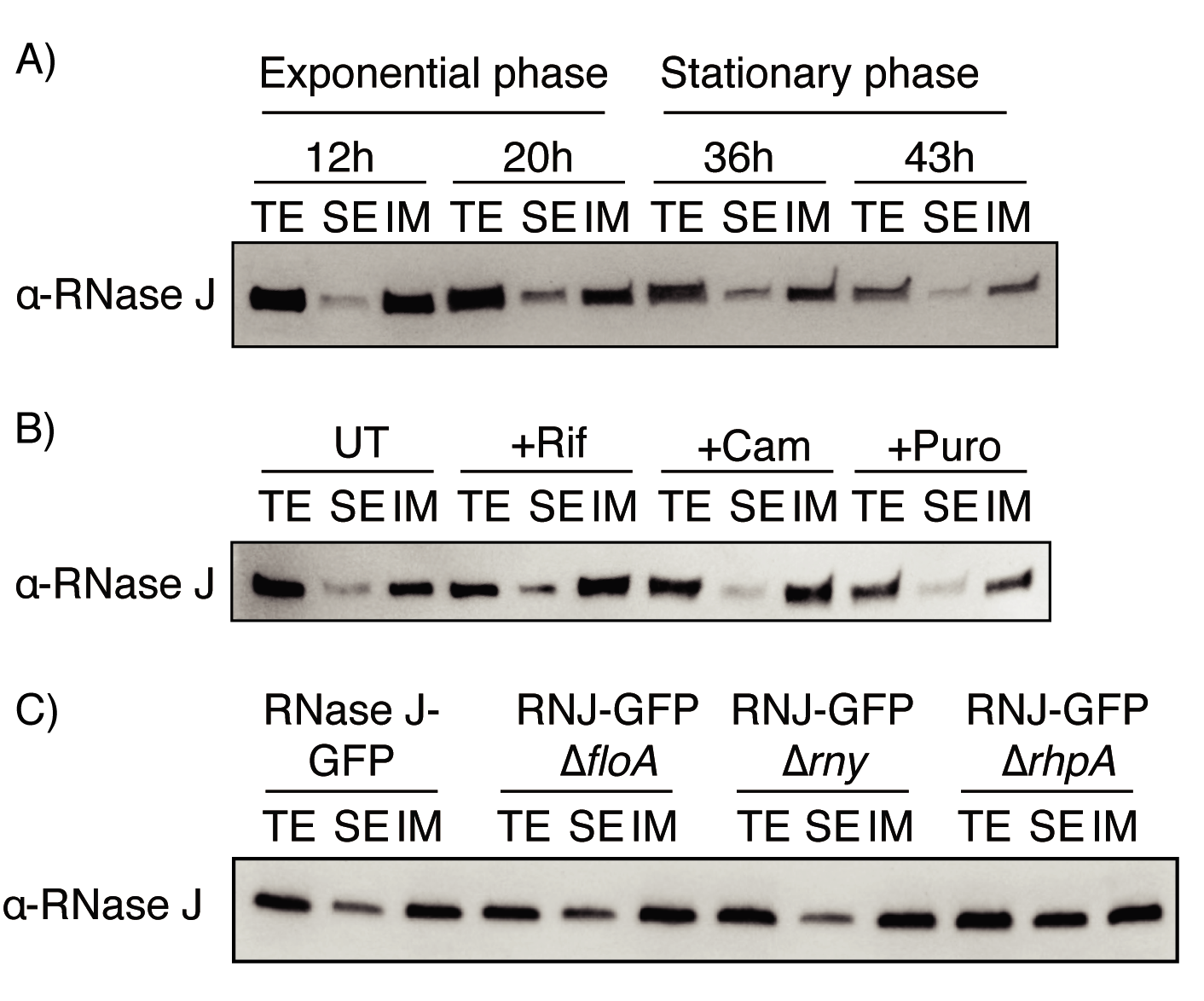

Supplement: FIG S3 [file mBio.01173-20-sf003.tif]
